# Determinants of human milk oligosaccharides profiles of participants in the STRONG kids 2 cohort

**DOI:** 10.3389/fnut.2023.1105668

**Published:** 2023-03-28

**Authors:** Yuting Fan, Anita Vinjamuri, Diane Tu, Carlito B. Lebrilla, Sharon M. Donovan

**Affiliations:** ^1^Department of Food Science and Human Nutrition, University of Illinois, Urbana, IL, United States; ^2^Department of Chemistry, University of California, Davis, Davis, CA, United States

**Keywords:** human milk oligosaccharides, secretor status, human milk, weight status, maternal intake

## Abstract

**Introduction:**

Human milk oligosaccharides (HMOS) are indigestible carbohydrates that support infant development by establishing a healthy microbiota, preventing infectious diseases, and promoting immune and cognitive development. Individual HMOS have distinct functions based on their chemical structures. HMO profiles can vary largely among mothers, but the research on factors other than genetic background affecting HMO composition are limited.

**Methods:**

In the present analysis, we examined the relationships between maternal characteristics and the HMO profiles of breastfeeding mothers (*n* = 392) in the STRONG kids 2 with the following demographic characteristics: average age: 30.8 y, 74.5% White, and 75.5% exclusively breastfeeding. Human milk samples were collected at 6 weeks postpartum and maternal information was obtained from self-reported surveys. Information on dietary intake changes since the participants have been breastfeeding was collected. HMO profiles were analyzed by high performance liquid chromatography coupled with mass spectrometry and secretor status was determined by the presence of four secretor markers [2′-fucosyllactose (2′-FL), LNFP I, LDFT, and TFLNH]. Spearmen correlation test was utilized to determine the relationships between individual HMOS and associations with maternal factors. Between-group differences in HMO relative abundances were examined with Kruskal-Wallis test.

**Results:**

Among all participants, 71.9% were secretors and 28.1% were non-secretors. The relative abundances of all HMOS differed (*p* < 0.05) by secretor status, with the exception for 6′-SL and 3′-SL. Positive correlations were observed among HMOS with similar structures, such as the 1,2-fucosylated HMOS. The abundances of selected HMOS were associated with maternal body weight, pregnancy complications, and dietary characteristics. Based on pre-pregnancy BMI, in all mothers, relative abundance of 3′-SL was significantly higher in overweight mothers than obese mothers (*p* = 0.013). In milk produced by non-secretor mothers, LNPF I + III abundances were greater in overweight than normal weight mothers (*p* = 0.020). Several HMO abundances were found to be associated with Gestational diabetes mellitus (GDM). Variations of HMO abundances were also observed with dietary food intake. In all mothers, egg consumption was positively correlated with LNT + LNnT (R = 0.13; *p* = 0.012) and cheese intake was positively associated with 2′-FL (R = 0.10; *p* = 0.046) and S-LNnH II (R = 0.11; *p* = 0.026) abundances.

**Discussion:**

HMO profiles were found to be associated with maternal characteristics and intake. Future research will investigate associations between HMOS and maternal and infant outcomes.

## 1. Introduction

Breastfeeding is recommended by numerous health organizations as the favored feeding method for early life because human milk (HM) contains nutrients and bioactive components that support infant development (1, 2). In particular, HM is considered distinctive for its high oligosaccharide content and complex structural composition (3–5). Human milk oligosaccharides (HMOS) are the third most abundant solid component in the HM (3–5); they facilitate the development of gut microbiota (6) by supporting the proliferation of healthy bacteria such as *Bifidobacterium longum* subsp. (7). LoCascio et al. reported that *Bifidobacterium longum* preferentially utilized certain shorter chain HMOS, suggesting that the prebiotic effect of HMOS could differ based on the relative composition of HMOS in HM (8). HMOS can also prevent infectious diseases by acting as soluble ligand analogs that block the binding of pathogens to intestinal epithelial cells (9–11). Aside from modulating intestinal health, HMOS have also been shown to impact infant immune system development (12–14) and are suggested to have effects on neurocognitive development in mammals (15, 16) and humans (17–20).

HMOS make up about 20% of all the carbohydrates presented in human breast milk in the amount of 5–15 g/L (21, 22). Analytical chemistry such as mass spectrometry has allowed the identification of HMO structures (23–25). Among the more than 200 individual HMOs identified and characterized to date (26), around 170 HMO chemical structures have been reported (27, 28). Generally, fewer than 20 structures account for more than 90% of the total HMO content of HM (29). The quantification of HMOS will be essential for understanding the presence of them in biological samples. The concentrations of the major HMOS have been determined in secretor and non-secretor donor’s milk at different stages of lactation (30). For example, average values of the concentrations of 2′-FL, 3-FL, LNT, 3′-SL, and 6′-SL have been calculated from the published data by weighted analysis (31). However, HMO profiles are highly variable between mothers within and across populations. Variations in HMOS content and composition can be potentially influenced by maternal genetics, biomedical conditions, environments, geographical locations, lifestyles, and time of lactation (29, 32, 33). However, the research on the impact of maternal characteristics on HMO profile variations is still somewhat limited.

As a component of our ongoing Synergistic Theory Research Obesity and Nutrition Group (STRONG) Kids 2 birth cohort study (34), the primary objective of this study was to investigate the associations between the maternal demographic information, pregnancy complications, dietary intake and HMO composition. We also examined the correlations among the individual HMOS and the influence of season when HM samples were collected on the HMO profile.

## 2. Methods

### 2.1. Study population

Study participants were selected from the STRONG Kids 2 cohort, which takes a cell to society approach to understand determinants of growth trajectories in early life and risk of childhood obesity (34). Pregnant mothers (*n* = 468) and their children were recruited from central Illinois area in the 3^rd^ trimester of pregnancy. Only mothers who were breastfeeding at 6 weeks and provided HM samples were included in the current analysis (*n* = 392). Mothers completed surveys regarding their demographic information including age, height, weight, and gestational age at delivery (Table 1). Mothers provided additional information regarding delivery methods, health history, and dietary changes during lactation (34). The STRONG kids 2 cohort study was approved by the University of Illinois Institution Review Board (#13448). Participants signed written consent in participating in the study.

**Table 1:** Demographic characteristics of the study participants.^1^

	Secretor (*n* = 282)	Non-secretor (*n* = 110)	Total (*n* = 392)
Age (years)	30.8 ± 4.40	31.0 ± 4.40	30.9 ± 4.40
Gestational age (weeks)	39.7 ± 1.15	39.6 ± 1.24	39.6 ± 1.17
Pre-pregnancy BMI	26.1 ± 6.28	26.9 ± 6.25	26.3 ± 6.28
Under weight	3 (1.1%)	2 (1.8%)	5 (1.3%)
Normal weight	141 (50.0%)	50 (45.5%)	191 (48.7%)
Overweight	74 (26.2%)	25 (22.7%)	99 (25.3%)
Obese	54 (19.1%)	30 (27.3%)	84 (21.4%)
Missing	10 (3.5%)	3 (2.7%)	13 (3.3%)
BMI at 6 week	27.8 ± 5.99	28.3 ± 5.78	27.9 ± 5.93
Normal weight	108 (38.3%)	38 (34.5%)	146 (37.2%)
Overweight	101 (35.8%)	37 (33.6%)	138 (35.2%)
Obese	71 (25.2%)	34 (30.9%)	105 (26.8%)
Missing	2(0.7%)	1 (0.9%)	3 (0.8%)
Race and ethnicity			
Non-hispanic white	290 (77.3%)	81 (29.4%)	290 (74.0%)
Non-hispanic non-white	42 (14.9%)	17 (15.5%)	59 (15.1%)
Hispanic white	8 (2.8%)	3 (2.7%)	11 (2.8%)
Hispanic non-white	2 (0.7%)	0 (0.0%)	2 (0.5%)
Did not report	21 (7.4%)	9 (8.2%)	30 (7.7%)
Feeding method			
Exclusive BF	210 (74.5%)	86 (78.2%)	296 (75.5%)
Mixed feeding	47 (16.7%)	14 (12.7%)	61 (15.6%)
Did not report^2^	25 (8.9%)	10 (9.1%)	35 (8.9%)
Developed PIH	12 (4.3%)	8 (7.3%)	20 (5.1%)
Developed GDM	18 (6.4%)	1 (0.9%)	19 (4.8%)

BMI, body mass index; BF, breastfeeding; PIH, Pregnancy Induced Hypertension; GDM, Gestational Diabetes Mellitus.

1 Values are means ± SD or *n* (%). There were no statistically significant demographic differences between secretor and non-secretor mothers.

2 Mother did not indicate whether they were exclusively or non-exclusively breastfeeding their infant.

### 2.2. HMO analysis

Human milk samples (*n* = 392) were collected at 6-weeks postpartum by complete breast expression by either hand or breast pump. The sample was mixed to homogeneity and a 30 ml aliquot removed and immediately placed in the home freezer. HM samples were collected from the home by the research staff, thawed, aliquoted and frozen at −80°C until sent to the laboratory of Dr. Carlito Lebrilla at the University of California, Davis for HMO analysis using established techniques (15). The HM samples were centrifuged to remove the lipid, and ethanol was used to precipitate out the protein in the milk. HMOS were reduced with sodium borohydride (Sigma-Aldrich, St. Louis, MO) and isolated with high-throughput solid-phase extraction on graphitized carbon cartridges (Glygen, Columbia, MD) to remove lactose and salts (32). All free HMOS were identified and annotated by comparing retention times and exact mass with an in-house library that was validated by high resolution HPLC-Chip/TOF mass spectrometry with either HMO standards or exoglycosidase digestion (35), and were expressed as relative abundances of the individual HMOS to the total oligosaccharide abundance in each HM sample (Table 2). Isomers that were unable to be chromatographically separated were grouped together for quantitation and named accordingly (35, 36). HMO composition varied in accordance with secretor genotypes, because secretor mothers have functional *FUT2* gene that enables the expression of α1,2 fucosyltransferase, which catalyzes the formation of α1,2-fucosylated compounds (37). Therefore, HMOS with Fuc α(1 → 2) Gal residues are considered secretor markers. FUT secretor status of the mothers were determined based on the abundance of four fucosylated structures (2′-FL, LNFP I, LDFT, and TFLNH) with a 6% threshold, because of their high sensitivity and specificity (32, 38). This method has been previously established and validated (38). Over 100 oligosaccharide structures were separated in each HM sample, and the 25 most abundant HMO structures were used in this study, which together accounted for >75% total HMO abundances on average across all samples.

**Table 2:** HMO relative abundance and diversity in the STRONG kids 2 cohort by secretor status.^1^

Retention time (Min)	HMO	Secretor (*n* =282)	Non-secretor (*n* = 110)	*p* value^3^
14.41	2′-FL	8.61 ± 2.73	0.23 ± 0.29	*p* < 0.01
2.70	3-FL	1.69 ± 1.27	4.82 ± 2.66	*p* < 0.01
20.42	3′-SL	0.10 ± 0.06	0.11 ± 0.07	N.S.
14.70	6′-SL	0.75 ± 0.43	0.71 ± 0.48	N.S.
16.01	LNT + LNnT	27.1 ± 4.66	36.1 ± 11.4	*p* < 0.01
16.08	LDFT	2.62 ± 1.71	0.03 ± 0.05	*p* < 0.01
15.95	LNFP V	0.73 ± 0.40	3.05 ± 1.21	*p* < 0.01
13.34	LNFP II	3.19 ± 1.80	7.31 ± 4.10	*p* < 0.01
15.62	LNFP I + III	15.2 ± 4.70	0.65 ± 0.41	*p* < 0.01
16.43	DFLNHa	1.61 ± 1.03	0.05 ± 0.05	*p* < 0.01
15.22	DFLNHb	1.84 ± 1.04	4.88 ± 2.23	*p* < 0.01
12.97	LNDFH I	4.24 ± 1.73	1.08 ± 0.62	*p* < 0.01
13.47	LNDFH II	0.41 ± 0.31	1.97 ± 1.19	*p* < 0.01
18.05	LNH	1.23 ± 0.64	1.05 ± 0.83	*p* < 0.05
18.47	LNnH	1.86 ± 1.21	0.65 ± 0.60	*p* < 0.01
20.46	p-LNH	0.25 ± 0.18	0.20 ± 0.37	*p* < 0.05
20.63	S-LNH	0.03 ± 0.03	0.01 ± 0.01	*p* < 0.01
21.48	S-LNnH II	1.16 ± 0.68	0.56 ± 0.54	*p* < 0.01
15.21	MFpLNH IV	0.47 ± 0.26	1.11 ± 0.52	*p* < 0.01
17.01	MFLNH I+ III	4.01 ± 1.39	6.20 ± 2.79	*p* < 0.01
17.66	IFLNH III	1.59 ± 0.72	1.41 ± 0.94	*p* < 0.05
19.94	IFLNH I	0.23 ± 0.21	0.03 ± 0.03	*p* < 0.01
20.66	DFS-LNH	0.13 ± 0.09	0.02 ± 0.02	*p* < 0.01
21.88	DFS-LNnH	0.10 ± 0.08	0.01 ± 0.01	*p* < 0.01
15.09	TFLNH	0.44 ± 0.26	0.07 ± 0.05	*p* < 0.01
-	HMO Diversity^2^	0.88 ± 0.03	0.83 ± 0.06	*p* < 0.01

HMO, human milk oligosaccharide; SD, standard deviation; 2′-FL, 2′-fucosyllactose; 3-FL, 3-fucosyllactose; 3′-SL, 3′-sialyllactose; 6′-SL, 6′-sialyllactose; LNnT, lacto-N-neotetraose; LNT, lacto-N-tetrose; LDFT, lactodifucotetraose; LNFP I/II/III/V, lacto-N-fucopentaose-I/II/III/V; DFLNHa/b, difucosyllacto-N-hexaose a/b; LNDFH I/II, lacto-N-difucohexaose I/II; LNH, lacto-N-hexaose; LNnH, lacto-N-neohexaose; p-LNH, para-lacto-N-hexaose; S-LNH, sialyl-lacto-N-hexaose; S-LNnH II, sialyllacto-*N*-neohexaose II; MFpLNH IV, fucosyl-para-lacto-*N*-hexaose IV; MFLNH I + III, Monofucosyllacto-*N*-hexaose I/III; IFLNH I/III, fucosyl-para-lacto-*N*-hexaose I/III; DFS-LNH, Difucosylmonosialyllacto-*N*-hexaose;DFS-LNnH, Difucosylmonosialyllacto-*N*-neohexaose; TFLNH, Trifucosyllacto-N-hexaose.

1 Values are means ± SD.

2 Calculated as the reciprocal sum of the square of the relative abundance of each individual HMO.

3 *p* value < 0.05 means the relative abundances of the HMO is significantly different by secretor status; N.S., not significant.

### 2.3. Exposure variables

Maternal age (years), gestational age at delivery (weeks), and health conditions including pregnancy induced hypertension (PIH) and gestational diabetes mellitus (GDM) were self-reported in the on-line surveys. Body mass index (BMI) pre-pregnancy and at the time of the milk collection (6 weeks postpartum) were calculated based on the reported height and weight at registration and measured height and weight at 6-weeks postpartum. Maternal weight category was defined as BMI < 18.5 for underweight (UW), BMI ≤ 24.9 for normal weight (NW), > 25 for overweight (OW), and > 30 for obese (OB). The season when the milk was collected was determined based on the calendar month of the HM collection, calculated according to the delivery date plus 6 weeks. Changes in maternal intake of selected food (egg, cheese, shellfish, other fish, milk, and yogurt), since the participants started breastfeeding were reported in the form of “did not eat before or now,” “eat about the same,” “eat less,” or “eat more.”

### 2.4. Statistical analysis

All statistical analyses were conducted using R (Version 4.1.1). For each secretor group, maternal characteristics were reported as means ± standard deviations (SD; Table 1). To determine HMO diversity, Simpson’s Diversity index (39) was calculated for each HMO, and the major HMO relative abundances and HMO diversity were compared between secretor groups with t tests. HMO profiles for all the participants and by secretor status were visualized with *ggplot*. Between HMO associations were analyzed with Nonparametric Spearman rank correlation and visualized with hierarchic heatmap (*ComplexHeatmap*). Spearman’s rho (ρ) was used to measure the strength of association between the individual HMO relative abundances. Kruskal-Wallis test were used for analyzing the HMO relative abundances differences between weight groups, race groups, pregnancy complication groups, and milk collection season, with Dunn Test as a post-hoc multiple comparison procedure. Analysis of covariances (ANCOVA) was used to adjust for other covariates include maternal age, gestational age in weeks, maternal BMI, and secretor status that could potentially associated with the HMO composition and diversity (transformed with boxcox transformation procedure). Pearson correlations were applied for correlations between HMO relative abundances and maternal pre-pregnancy BMI and BMI at 6 weeks postpartum. Changes in maternal dietary intake during pregnancy were coded as: (1) eat less; (2) do not eat before or now; (3) eat about the same; and (4) eat more since breastfeeding compared to before, and Spearman rank correlation test was performed with the log transformed value. Significance of associations was set at *p* ≤ 0.05 for all analyses. Multiple comparisons were adjusted with Bonferroni correction procedure of 0.05 Type I error rate.

## 3. Results

### 3.1. Participant demographics

Study participants were 30.8 ± 4.4 years of age, and had a gestational age of at delivery of 39.6 ± 1.17 weeks. The majority of the participants were white (74.5%). Most of the participants were exclusively breastfeeding their infants at 6 weeks (75.5%), whereas the remainder were either supplementing with formula (15.6%) or did not report their mode of feeding. Participant pre-pregnancy BMI was 26.3 ± 6.28; and BMI at the time of HM collection was 27.9 ± 5.93. Twenty participants reported PIH and 19 participants reported GDM (Table 1). There were no significant demographic differences between secretor and non-secretor mothers.

### 3.2. HMO profiles

**Figure 1:**
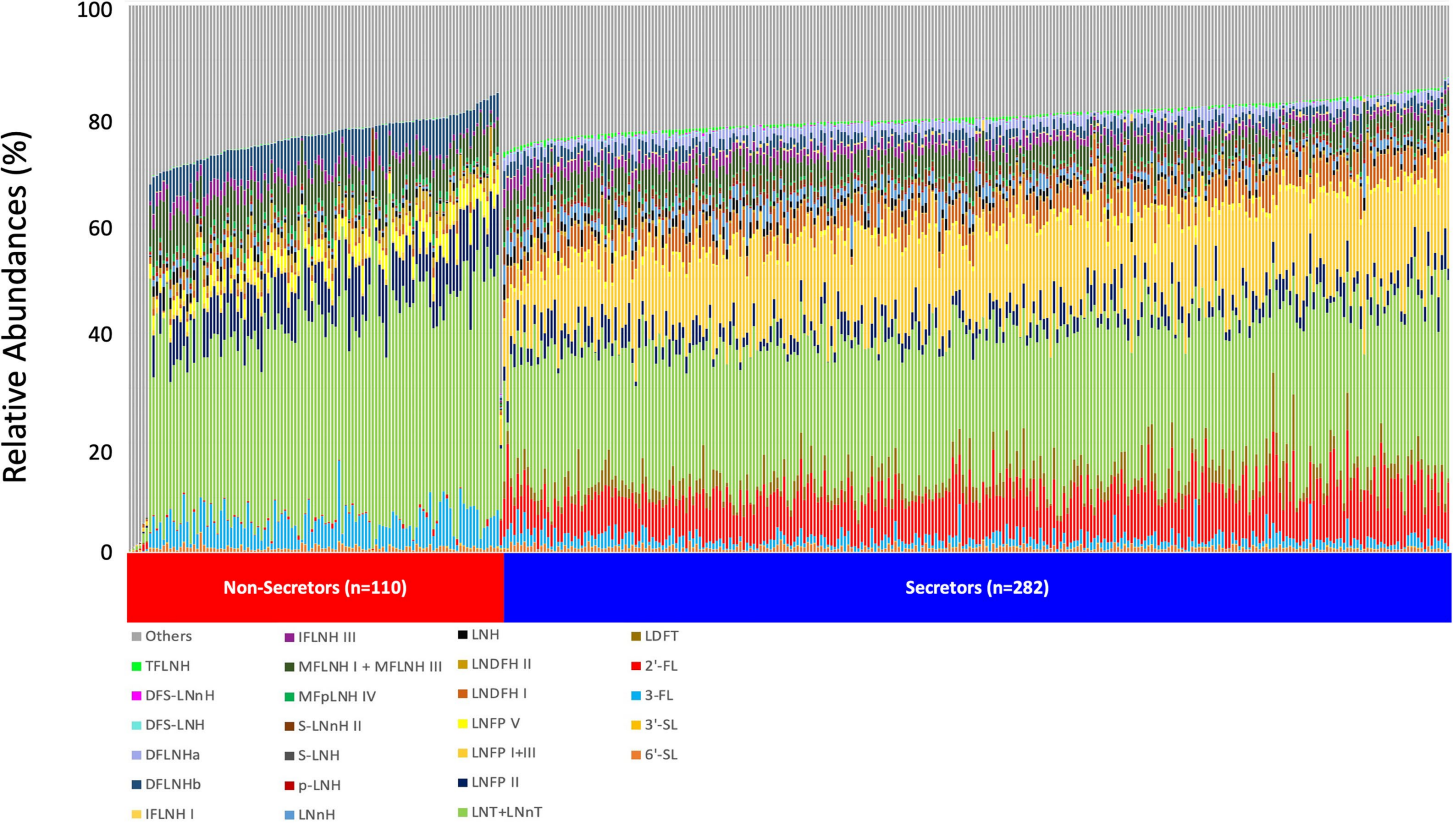
HMO relative abundance among 392 participants in the STRONG Kids 2 cohort. Boxes indicate IQRs; Solid lines indicate medians; whiskers indicate ranges (IQRs) excluding the outliers; dots indicates outliers. HMO, human milk oligosaccharide; 2′-FL, 2′-fucosyllactose; 3-FL, 3-fucosyllactose; 3′-SL, 3′-sialyllactose; 6′-SL, 6′-sialyllactose; LNnT, lacto-N-neotetraose; LNT, lacto-N-tetrose; LDFT, lactodifucotetraose; LNFP I/II/III/V, lacto-N-fucopentaose-I/II/III/V; DFLNHa/b, difucosyllacto-N-hexaose a/b; LNDFH I/II, lacto-N-difucohexaose I/II; LNH, lacto-N-hexaose; LNnH, lacto-N-neohexaose; *p*-LNH, *para*-lacto-*N*-hexaose; S-LNH, sialyl-lacto-*N*-hexaose; S-LNnH II, sialyllacto-*N*-neohexaose II; MFpLNH IV, fucosyl-*para*-lacto-*N*-hexaose IV; MFLNH I/III, monofucosyllacto-*N*-hexaose I/III; IFLNH I/III, fucosyl-*para*-lacto-*N*-hexaose I + III; DFS-LNH, difucosylmonosialyllacto-*N*-hexaose;DFS-LNnH, difucosylmonosialyllacto-*N*-neohexaose; TFLNH, trifucosyllacto-*N*-hexaose.

**Figure 2:**
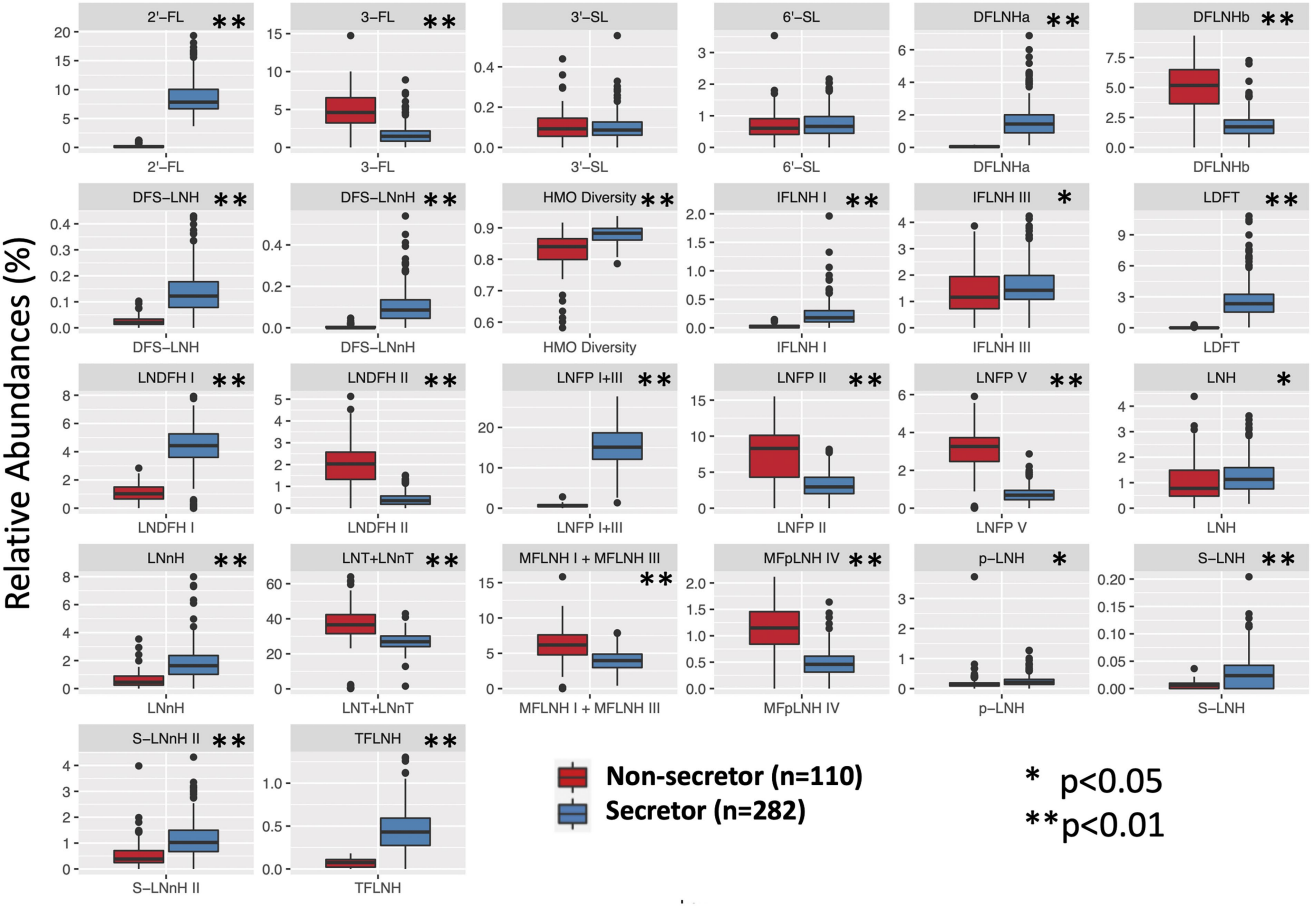
HMO relative abundance among 392 participants in the STRONG Kids 2 cohort by secretor status. S, secretors; NS, non-secretors. Boxes indicate IQRs; Solid lines indicate medians; whiskers indicate ranges (IQRs) excluding the outliers; dots indicates outliers. HMO, human milk oligosaccharide; 2′-FL, 2′-fucosyllactose; 3-FL, 3-fucosyllactose; 3′-SL, 3′-sialyllactose; 6′-SL, 6′-sialyllactose; LNnT, lacto-N-neotetraose; LNT, lacto-N-tetrose; LDFT, lactodifucotetraose; LNFP I/II/III/V, lacto-N-fucopentaose-I/II/III/V; DFLNHa/b, difucosyllacto-N-hexaose a/b; LNDFH I/II, lacto-N-difucohexaose I/II; LNH, lacto-N-hexaose; LNnH, lacto-N-neohexaose; *p-*LNH, *para*-lacto-*N*-hexaose; S-LNH, sialyl-lacto-*N*-hexaose; S-LNnH II, sialyllacto-*N*-neohexaose II; MFpLNH IV, fucosyl-*para*-lacto-*N*-hexaose IV; MFLNH I/III, monofucosyllacto-*N*-hexaose I/III; IFLNH I/III, fucosyl-*para*-lacto-*N*-hexaose I + III; DFS-LNH, difucosylmonosialyllacto-*N*-hexaose;DFS-LNnH, difucosylmonosialyllacto-*N*-neohexaose; TFLNH, trifucosyllacto-*N*-hexaose.

The relative abundance of 25 individual HMOS in highest abundances were included in the association analysis (Figure 1). Among the 392 participants, 282 (71.9%) mothers were secretors and 110 (28.1%) were non-secretors. Secretor mothers had a higher (*p* < 0.001) HMO diversity index (0.88 ± 0.03) compared with the non-secretor mothers (0.83 ± 0.06). With the exception of 6′-SL and 3′-SL, all other HMO relative abundances differed by secretor status (Table 2; Figure 2). Abundances of *FUT-2* dependent HMOs (2′-FL and LNFP I + III, LDFT, and TFLNH), DFLNHa, LNDFH I, DFS-LNH, DFS-LNnH, IFLNH I, IFLNH III, LNH, LNnH, p-LNH, S-LNH, and S-LNnH II, are significantly higher in HM produced by secretor mothers (all *p* ≤ 0.05). Other HMOS were higher in HM from non-secretor mothers, including 3-FL, DFLNHb, LNDFH II, LNFP V, LNFP II, LNT + LNnT, MFLNH I + MFLNH III, and MFpLNH IV (all *p* ≤ 0.01; Table 2; Figure 2).

### 3.3. Correlations between HMOS

**Figure 3:**
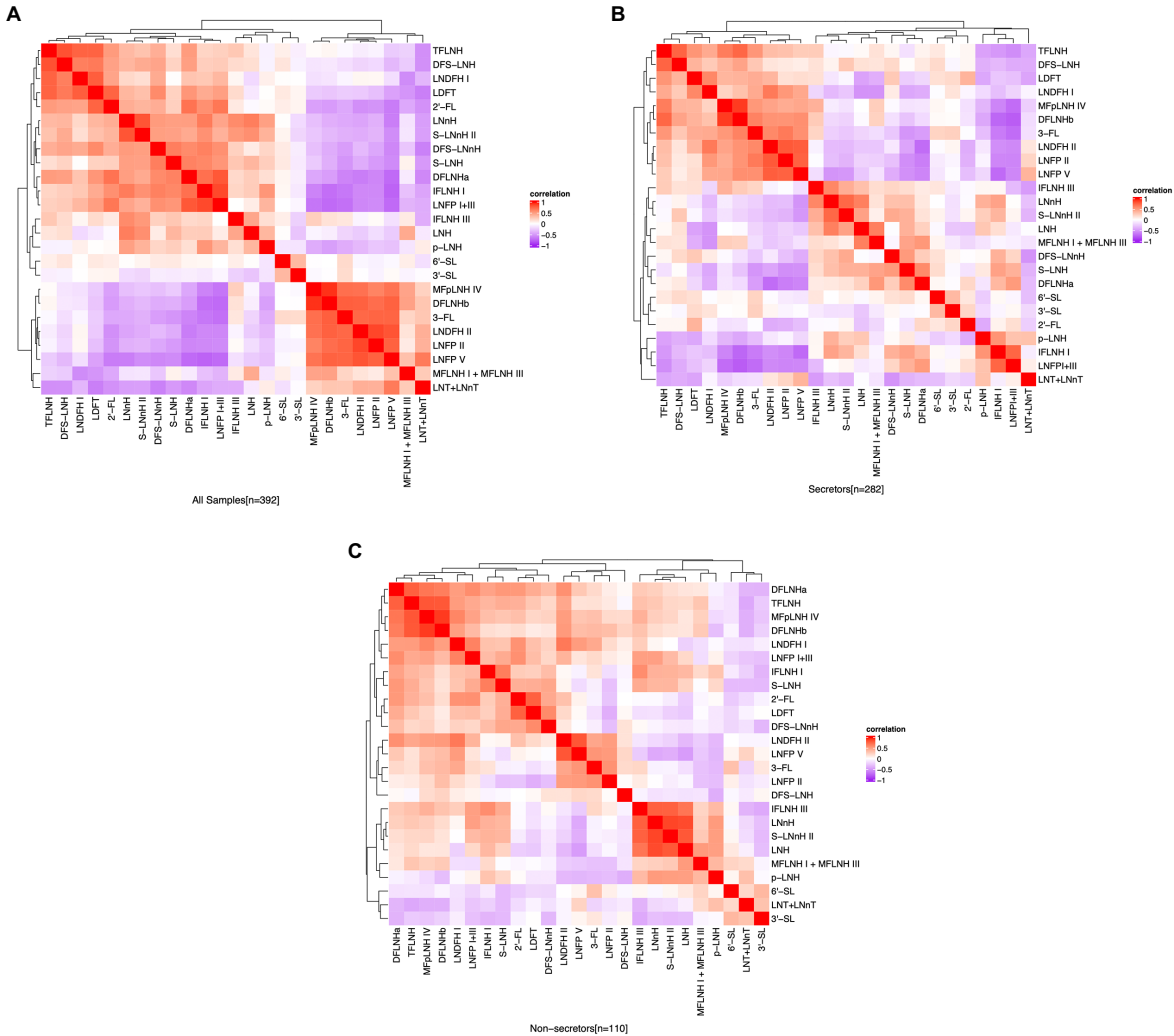
Correlation between HMO relative abundance among the participants in the STRONG Kids 2 cohort, all participants **(A)**, secretors **(B)**, and non-secretors **(C)**. Color reflect the direction and strength of the Spearman correlations between individual HMOs. S, secretors; NS, non-secretors; HMO, human milk oligosaccharide; 2′-FL, 2′-fucosyllactose; 3-FL, 3-fucosyllactose; 3′-SL, 3′-sialyllactose; 6′-SL, 6′-sialyllactose; LNnT, lacto-N-neotetraose; LNT, lacto-N-tetrose; LDFT, lactodifucotetraose; LNFP I/II/III/V, lacto-N-fucopentaose-I/II/III/V; DFLNHa/b, difucosyllacto-N-hexaose a/b; LNDFH I/II, lacto-N-difucohexaose I/II; LNH, lacto-N-hexaose; LNnH, lacto-N-neohexaose; *p-*LNH, *para*-lacto-*N*-hexaose; S-LNH, sialyl-lacto-*N*-hexaose; S-LNnH II, sialyllacto-*N*-neohexaose II; MFpLNH IV, fucosyl-*para*-lacto-*N*-hexaose IV; MFLNH I/III, monofucosyllacto-*N*-hexaose I/III; IFLNH I/III, fucosyl-*para*-lacto-*N*-hexaose I + III; DFS-LNH, difucosylmonosialyllacto-*N*-hexaose;DFS-LNnH, difucosylmonosialyllacto-*N*-neohexaose; TFLNH, trifucosyllacto-*N*-hexaose.

The relative abundance of 2′-FL was positively correlated (*p* ≤ 0.05) with the abundances of LDFT (ρ = 0.76), LNFP I + III (ρ = 0.68), LNDFH I (ρ = 0.47), and DFLNHa (ρ = 0.70). The relative abundance of 3-FL was positively correlated with LNFP V (ρ = 0.76), LNFP II (ρ = 0.76), LNDFH II (ρ = 0.75), and DFLNHb (ρ = 0.78). Among all the participants, 2′-FL was negatively correlated with 3-FL (ρ = −0.43), LNT + LNnT (ρ = −0.54), LNFP V (ρ = −0.65), LNFP II (ρ = −0.53), DFLNHb (ρ = −0.50), and LNDFH II (ρ = −0.56; Figure 3A). Additionally, there were some distinct between-HMO correlations based on secretor status. For example, in HM of secretor mothers, 2′-FL and 6′-SL was negatively correlated with p-LNH (ρ = −0.42) and (ρ = −0.25, respectively; Figure 3B), but these relationships were not observed in the HM of non-secretors (Figure 3C).

### 3.4. Correlations between HMOS and maternal characteristics

The HMOs were first grouped into “total fucosylated,” “total sialylated” and “neutral” based on similar chemical structures, and the relative abundances of the groups of HMOs were summed for the analysis with maternal characteristics. However, no significant associations were found for the grouped HMO; thus associations for individual HMO structures were investigated.

When HM samples were considered *independent of secretor status*, there were significant differences in 3′-SL relative abundance in HM collected from mothers with different weight status within all samples based on pre-pregnancy BMI. Specifically, milk from OW group (0.11 ± 0.07) was higher in 3′-SL compared to milk from OB mothers (0.09 ± 0.07; *p* = 0.013). There were no differences in HMOS relative abundances among the different weight groups based on 6 weeks postpartum BMI.

However, several HMOS differed by weight groups dependent on the secretor status. In non-secretor HM, 3-FL abundances were significantly higher (*p* = 0.036) in NW group (5.33 ± 2.11) than the OB group (3.78 ± 2.25) pre-pregnancy. LNFP I + III abundance in the OW group (0.75 ± 0.38) was significantly higher than in NW group (0.56 ± 0.48; *p* = 0.020) based on BMI at 6 weeks. Those relationships remained significant after adjusting for maternal age and gestational age. When comparing mothers with different weight categories, no significant variations in HMO diversity and individual HMO abundances were found either for weight status pre-pregnancy or at week 6.

Pearson correlation analysis was applied to determine the relationships between maternal BMI and HMO relative abundances and diversity. Negative relationships were found between pre-pregnancy BMI and the relative abundances of 3-FL (R = −0.22; *p* = 0.022) and LNFP V (R = −0.20, *p* = 0.038), but those correlations were only found in non-secretor mothers and not in the secretor mothers. Some relationships between HMOS concentration and maternal age were observed. Higher maternal age was associated with greater relative abundances of 3-FL (R = 0.12, *p* = 0.019), DFLNHb (R = 0.11, *p* = 0.034), and IFLNH III (R = 0.1, *p* = 0.046), and lower abundances of p-LNH (R = −0.13, *p* = 0.011) and IFLNH I (R = −0.13, *p* = 0.012). In addition, maternal age was negatively associated with gestational age of the mothers (R = −0.11, *p* = 0.034). There was no observed effect of season of HM collection on HMOS profiles.

Participants who were identified as White had significantly greater relative abundances of LNFP II (*p* = 0.004) and TFLNH (*p* = 0.008) compared to non-White participants, independent of ethnicity. The relationships remained statistically significant after adjusting for maternal BMI, maternal age, and secretor status.

Eight HMOS differed between mothers who developed GDM and those that did not (Table 3). Mothers who developed GDM had higher LDFT (*p* = 0.013), LNDFH I (*p* = 0.014), LNnH (*p* = 0.024), S-LNnH II (*p* = 0.020), and DFS-LNH (*p* = 0.030) relative abundances compared to mothers who did not have GDM. In contrast, mothers with GDM had lower relative abundances of MFLNH I + III (*p* = 0.004), MFpLNH IV (*p* = 0.005), and DFLNHb (*p* = 0.010) than mothers who did not have GDM. No statistically significant differences were found in HMOS relative abundances between participants with PIH or without PIH.

**Table 3:** Relative abundance of HMOS that differed between STRONG Kids 2 participants who developed Gestational Diabetes Mellitus.

	Developed GDM (*n* = 19)	Did not develop GDM (*n* = 373)	*p* value
LDFT	2.94 ± 2.44	1.84 ± 1.82	0.013
LNDFH I	4.51 ± 1.73	3.30 ± 2.07	0.014
LNnH	2.13 ± 1.65	1.49 ± 1.17	0.024
S-LNnH II	1.24 ± 0.64	0.98 ± 0.70	0.020
DFS-LNH	0.13 ± 0.06	0.10 ± 0.09	0.030
MFpLNH I + III	3.33 ± 1.53	4.69 ± 2.14	0.004
MFLNH IV	0.38 ± 0.23	0.66 ± 0.45	0.005
DFLNHb	1.56 ± 0.76	2.75 ± 2.03	0.010

### 3.5. Correlations between HMOS and maternal food intake

There were no significant correlations between changes in maternal intake and HMO diversity when all HM samples were considered. For several HMOS, significant associations were discovered with changes in maternal food intake during lactation, and those influences of food intake varied based on secretor status.

**Figure 4:**
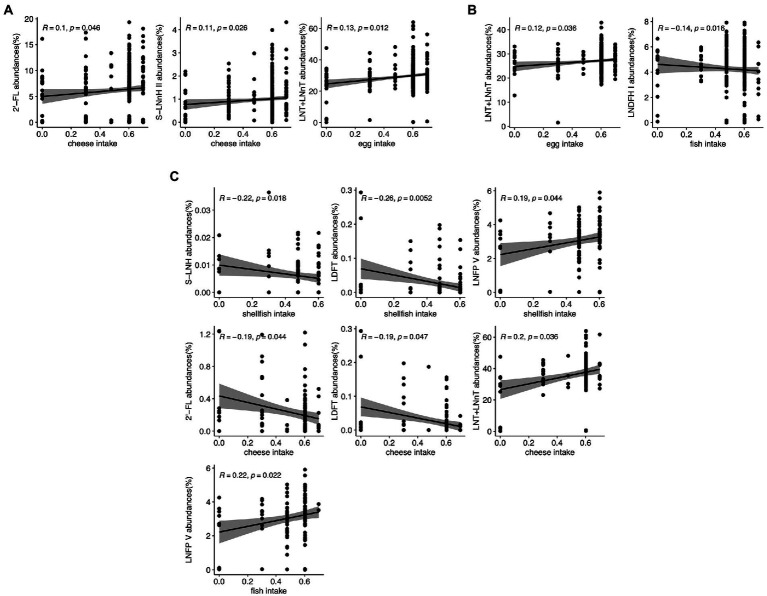
Correlation between maternal food intake and individual HMO relative abundance in the STRONG Kids 2 cohort in all participants **(A)**, Secretors **(B)**, and Non-secretors **(C)**. R value reflects the strength of Spearman correlation. 2′-FL, 2′-fucosyllactose; LNnT, lacto-N-neotetraose; LNT, lacto-N-tetrose; LDFT, lactodifucotetraose; LNFP I/II/III/V, lacto-N-fucopentaose-I/II/III/V; S-LNnH II, sialyllacto-*N*-neohexaose II.

In the full cohort, higher cheese consumption was correlated with greater amount of 2′-FL and S-LNnH II (*p* = 0.046 and 0.026, respectively). Increased egg consumption was correlated with greater LNT + LNnT abundance (*p* = 0.012; Figure 4A). In secretor mothers, the positive association between change in egg intake and LNT + LNnT abundances remained significant (*p* = 0.036), and fish intake was negatively associated with LNDFH I abundances (*p* = 0.016; Figure 4B). HM from non-secretor mothers showed more associations between dietary intake changes and HMO abundances: shellfish intake was negatively correlated with LDFT and S-LNH abundances (*p* = 0.0052 and 0.018, respectively), but positively correlated with LNFP V abundances (*p* = 0.044). Cheese intake was negatively associated with 2′-FL and LDFT abundances (*p* = 0.044 and 0.047, respectively), but positively correlated with LNT + LNnT (*p* = 0.036). Lastly, fish consumption by non-secretor mothers was positively associated with LNFP V (*p* = 0.022; Figure 4C).

## 4. Discussion

Human milk collected at 6-weeks postpartum from participants in the STRONG kids 2 cohort showed high variability in the relative abundances of specific HMOS within and between secretor status. The HMO profiles variations within secretor status suggested the relative abundances and compositions of HMOS are impacted by factors beyond maternal genetic background. In addition, significant associations were found between HMOS relative abundances and maternal weight status, age, race, health status, and food intake in our study population.

The content and composition of HMOS are related to genetic background, Lewis blood type and secretor status of the mothers (40), and variations of HMO profile in the STRONG kids 2 participants were observed between the secretor groups as determined by the secretor markers. HMO diversity was significantly higher for the secretor mothers compared to the non-secretor mothers, who do not synthesize α1,2-fucosylated HMOS, resulting in less diverse HMO profile. Approximately 15–20% of women worldwide do not express *FUT2* gene and are considered non-secretors (41). In general, the proportion of non-secretors is lower than those classified as secretors. However, the proportion of non-secretors varies by country, ranging from 0% in Bolivia to up to 37% in South Africa and 36% in the Gambia (32). Additionally, the proportion of secretor and non-secretor mothers can be quite different in populations within the United States (22). For example, a population of women located in Washington state, with unspecified ethnicity, had 32% non-secretors, whereas another group of lactating women in California, who self-reported to be Hispanic, had only 5% non-secretors. The STRONG kids 2 cohort is located in central Illinois in the United States (34) and consists of predominantly white, non-Hispanic women, had a relatively high percentage of non-secretor (28.1%) mothers compared to the global average (15).

Correlations between individual HMOS support relationships among HMOS synthesized along common elongation pathways. For example, 2′-FL relative abundance was positively correlated with the relative abundances of LDFT, LNFP I + III, LNDFH I, and DFLNHa, which was expected because they all have α1,2-fucose linked to terminal galactose (42). Although LNFP III should not be included in this category, we were not able to distinguish LNFP I and LNFP III chromatographically during HMO extraction and identification and, thus, their relative abundances were added together. Also, the negative correlation between 2′-FL and 3-FL could potentially result from *FUT2* and *FUT3* enzymes competing for some of the same substrates (22).

Most of the individual HMO relative abundances differed by secretor status, except for 3′-SL and 6′-SL. These sialylated HMOS clustered together and were positively correlated with each other. Neutral HMOS, LNH, p-LNH, and IFLNH III, clustered together and were positively correlated with each other. In non-secretors, LNT + LNnT, DFLNHb, LNDFH II, LNFP V, LNFP II, MFpLNH I + III relative abundances were significantly greater than secretors; Similar results were observed in the Maternal and Infants Nutrition Cohort Study cohort in China (43), which compared the HMOS concentrations in secretor groups, with the exception of LNFP V.

In terms of maternal characteristics, our results are consistent with previous research showing that maternal weight status was associated with the relative abundance of specific HMOS. Isganaitis et al. reported that maternal prepregnancy BMI was linked to differences in several HMO structures, including LNFP I, LNFP I + III, and 2′-FL in a study of 35 mother-infant pairs (44). Similarly, a positive correlation between maternal BMI and 2′-FL concentrations was also reported by Tonon et al. in 78 Brazilian women (45). Nonetheless, several other studies reported no significant association between maternal weight status and HMO concentrations. For instance, Oliveros et al. found that 6′-SL and 2′-FL were not influenced by preconceptional BMI or the development of GDM (18), and Berger et al. pointed out that prepregnancy BMI was not associated with any of the 19 HMOs, including 2′-FL at 1 and 6 months of infant age (46).

Besides, the analysis of 194 mother–child pairs at the Arkansas Children’s Nutrition Center (47) revealed that maternal BMI was a positive predictor for the concentrations (μg/ml) of LNnT, 3-FL and 6′-SL in HM collected at 2 months postpartum. McGuire and co-workers also reported associations between maternal postpartum BMI and HMOS in their HM samples collected between 2 weeks and 5 months postpartum; A positive association between concentrations of 2′-FL and maternal BMI was reported, whereas DSLNT, a sialylated HMO, was inversely correlated with maternal BMI (48). Saben and colleagues hypothesized that maternal weight status might be negatively associated with HMO sialylation as well; lower concentrations of sialylated HMOS at 2 months after delivery, including DSLNT, DSLNH, and FDSLNH, were found in overweight mothers (47). However, this relationship was not observed in our data. In our study, maternal pre-pregnancy BMI was only found to be negatively associated with 3-FL and LNFP V in HM collected 6 weeks postpartum and only in non-secretor mothers.

When looking at weight groups of the mothers, Saben et al. (47) also pointed out that secretor mothers who were overweight had lower absolute concentration of LNT and LNnT than secretor women with normal weight and obesity. In our study, we did not discover significant differences between weight groups; while we observed negative correlation between pre-pregnancy BMI and LNT + LNnT relative abundances (R = −0.15), but the negative correlation did not reach statistical significance. Samuel and colleagues (49) also observed that European women (*n* = 290) with a pre-pregnancy BMI in the overweight category had lower concentrations of LNnT at day 2 and LNT at days 30 and 90 postpartum than normal weight mothers, after adjusting for secretor status. Although the associations were observed in different populations at different stages of lactation, our results were consistent with other research findings, suggesting that maternal overweight is associated with lower LNT and LNnT abundances and concentrations. Additionally, in the Arkansas Children’s Nutrition Center cohort, 3′-SL concentrations were significantly lower in non-secretor mothers with overweight women compared to both normal weight and women with obesity in based on pre-pregnancy BMI, while an opposite relationship was observed in our full cohort when comparing the weight groups based on pre-pregnancy BMI.

We also report that LNFP I + III abundance was significantly higher in the HM of non-secretor women in the OW group compared to the NW group based on BMI at 6 weeks, which had not been reported previously. It should be noted that not all studies have reported associations between maternal weight status and HMOS. For example, no significant associations between maternal BMI and HMOS composition were observed at 3–4 months postpartum in a subset (*n* = 427) of participants in the Canadian CHILD cohort (29). In addition, it has been noted that maternal BMI during the immediate postpartum period might not be a best indicator of maternal adiposity (22). Thus, to investigate the relationship between maternal adiposity and HMO relative concentration, better techniques are needed to investigate body composition more accurately (48). Several associations were found between maternal age and HMOS abundance. For instance, a significant positive correlation between maternal age and DFLNHb abundance was revealed in our analysis, which was also seen in the Maternal and Infants Nutrition Cohort Study (43). In the CHILD cohort, a negative association between maternal age and DFLNT concentration was found (29). Ferreira et al. only observed significant correlation between maternal age and FLNH in a sensitivity analyses in a *n* = 15 subset of a cohort from Brazil at 28–50 and 88–119 days postpartum (50). Negative correlation between maternal age and sialylated LSTc concentrations was seen in Chinese mothers (*n* = 110) (51). In the current study, higher maternal age was associated with lower abundances of p-LNH and IFLNH I. Some other studies did not find any associations between HMO levels and maternal age (52). Thus, no consistent relationship between maternal age and individual HMOS has been observed, although fucosylated HMOS were more likely to be positively associated with maternal age.

In addition, a prospective study conducted in Toronto, Canada reported that race was a strong predictor of HMO concentrations among non-secretor mothers, where a higher concentration of LNFP II was observed among White mothers compared to other races (53). A similar relationship was observed in all samples in our cohort, where greater relative abundances of LNFP II (*p* = 0.004) was found compared to non-white mothers. Although it has been shown that there is a relationship between secretor status and race, the statistical significance remained after adjusting for secretor status.

Only one study reported associations between HMO profile and glucose metabolism: In a study of 87 participants recruited through midwife practices and hospitals in Amsterdam, significantly greater 3′-SL concentrations at 15 and 24 weeks postpartum were in found in women who had developed GDM in pregnancy (33). This study also reported that LDFT in early pregnancy was associated with higher fasting insulin and insulin resistance at 24 weeks postpartum (54). In the current study, significantly higher LDFT relative abundances at 6 weeks postpartum was uncovered in participants developed GDM, suggesting the possible association between LDFT abundances and insulin resistance.

Azad and colleagues reported no major differences in HMO concentrations by season of HM collection, with only LNFP III being significantly lower in HM collected in winter or spring in Canadian women participating in the CHILD cohort (29). Interestingly, in our study, LNFP III was also found to be numerically lowest in HM collected in spring compared to the other three seasons, but that difference did not reach statistical significance. No significant differences in the relative abundances of individual HMOS were observed based on season of milk collection, even after adjusting for secretor status. A study conducted in the Gambia (55) reported that higher concentrations of HMOS were observed in the dry season than during the wet season. However, this relationship was likely to be influenced by dietary intake, since food availability is more limited during the wet season (31). Thus, we were interested in investigating whether changes in maternal dietary intake was associated with HMOS.

The literature examining relationships between maternal dietary intake and HMOS is somewhat limited. Several studies have investigated overall dietary patterns (33, 34), whereas other investigated relationships with specific dietary components (31, 32). Azad and coworkers (13) utilized the Healthy Eating Index (HEI), which measures diet quality based on adherence to the United States Dietary Guidelines for Americans (56) to investigate whether maternal intake during pregnancy was associated with HMOS, but no clear evidence of its influence on HMOS profile was uncovered. Similarly, Neville and coworkers concluded that dietary patterns did not result in different HMO profiles among the vegan (*n* = 26), vegetarian (*n* = 22), and nonvegetarian (*n* = 26) lactating women (57). Williams and colleagues investigated the associations between the nutrient components in the HM and the HMO composition and several significances were uncovered in a small sample (*n* = 16) of breastfeeding women. For example, myristoleic acid was positively correlated with the absolute concentration of LNT and LNFP I, and stearic acid was negatively correlated with the total HMO concentration. However, it is unclear that whether maternal dietary intake had an impact on the interaction between HM lipids and HMO profile, (58), although the fatty acid composition of HM can be influenced by maternal diet (59).

Lipid-based supplementation during pregnancy were not correlated with HMO concentrations (60). However, an observational study conducted in Shanxi, China, found a positive association between vitamin A intake as determined by 72-h dietary recall and sialylated HMOS in the HM collected at day 40 of lactation in 90 healthy women (32). Additionally, a probiotic supplementation study conducted in Helsinki, Finland of 1,223 pregnant mothers discovered that those who were supplemented with probiotics showed higher concentrations of 3-FL and 3′-SL (61).Thus, most of the studies did not reveal significant associations between maternal intake during pregnancy and HMO profile. Given that HMO relative abundances and concentrations can vary across lactation stages (29), it might be practical to examine changes in maternal intake during lactation.

In the STRONG kids 2 cohort, we assessed longitudinal dietary intakes by food frequency questionnaires (FFQ), however, the earliest FFQ was not collected until 3 months postpartum. Since direct measures of maternal dietary intake at the time of HM collection (6 weeks) were not available, we investigated whether recent reported changes in maternal dietary intake during lactation. In the STRONG kids2 cohort, we identified several correlations between changes in the intake of selected protein-rich foods during lactation and the relative abundance of individual HMOS. For example, an increase in shellfish intake was associated with lower LDFT abundances in all samples and increased consumption of other fish was positively associated with LNFP V abundance in non-secretor mothers. Egg intake was positively associated with LNT + LNnT relative abundances in secretor mothers.

Strengths of the current study include its relatively large sample size and the observed associations between maternal food intake changes during lactation and HMO abundances. However, there are some limitations in our study as well. For example, most maternal factors were associated with only a few individual HMO structures, and the HMOS were expressed as relative abundances instead of absolute quantification. In addition, maternal dietary intake information was obtained through self-reported survey, and the data was expressed as the change of intake since breastfeeding compared to their usual intake. While we do not have data available for their usual intake, the change in food intake may not accurately define the absolute amount of food consumption, and it must be further investigated and confirmed by future studies.

In summary, this study provides information regarding associations between maternal characteristics, including secretor status, weight categories, maternal age, and dietary intake, and the HMO profile variations. Among the maternal factors shown to be significantly associated with HMO relative abundances, genetic background of the mothers was the most important factor contributing to the HMO profile differences, which supports previous works that also looked at maternal determinants of HMO profiles. Most of the factors associated with HMO profiles that were not directly linked to maternal genetics require further research in order to determine implications of mothers’ health status on infants because HMO composition may influence diseases susceptibility, growth and development (62, 63). Especially, maternal dietary intake changes during lactation needs to be considered as a potential factor affecting HMO composition in future breast milk studies.

## Data availability statement

The raw data supporting the conclusions of this article will be made available by the authors, without undue reservation.

## Ethics statement

The studies involving human participants were reviewed and approved by Institutional Review Board, University of Illinois, Urbana-Champaign. The patients/participants provided their written informed consent to participate in this study.

## Author contributions

SD contributed to the conception and design of the study and obtained research funding. YF, AV, and DT conducted the HMO analyses. SD and CL contributed to the interpretation of the data. YF performed the statistical analyses and wrote the manuscript. All authors contributed to the article and approved the submitted version.

## Funding

This research was funded by the National Dairy Council, the National Institutes of Health (NIH R01 DK107561), the Gerber Foundation, and USDA Hatch funding. YF is supported by the Jeanette Chu and Winston Y. Lo Endowed Fellowship from the Department of Food Science and Human Nutrition at the University of Illinois, Urbana-Champaign and USDA Hatch funding (Illu-6980979).

## Conflict of interest

The authors declare that the research was conducted in the absence of any commercial or financial relationships that could be construed as a potential conflict of interest.

## Publisher’s note

All claims expressed in this article are solely those of the authors and do not necessarily represent those of their affiliated organizations, or those of the publisher, the editors and the reviewers. Any product that may be evaluated in this article, or claim that may be made by its manufacturer, is not guaranteed or endorsed by the publisher.
